# Physician perceptions of pharmacist roles in a primary care setting in Qatar

**DOI:** 10.1186/1744-8603-8-12

**Published:** 2012-05-31

**Authors:** Kerry Wilbur, Amina Beniles, Arwa Hammuda

**Affiliations:** 1 College of Pharmacy, Qatar University, PO Box 2713, Doha, Qatar

**Keywords:** Physician, Perceptions, Pharmacists, Qatar

## Abstract

**Purpose:**

Pharmacists are uniquely trained to provide guidance to patients in the selection of appropriate non-prescription therapy. Physicians in Qatar may not always recognize how pharmacists function in assuring safe medication use. Both these health professional groups come from heterogeneous training and experiences before migrating to the country and these backgrounds could influence collaborative patient care. Qatar Petroleum (QP), the largest private employer in the country, has developed a pharmacist-guided medication consulting service at their primary care clinics, but physician comfort with pharmacists recommending drug therapy is currently unknown. The objective of this study is to characterize physician perceptions of pharmacists and their roles in a primary care patient setting in Qatar.

**Methods:**

This cross-sectional survey was developed following a comprehensive literature review and administered in English and Arabic. Consenting QP physicians were asked questions to assess experiences, comfort and expectations of pharmacist roles and abilities to provide medication-related advice and recommend and monitor therapies.

**Results:**

The median age of the 62 (77.5%) physicians who responded was between 40 and 50 years old and almost two-third were men (64.5%). Fourteen different nationalities were represented. Physicians were more comfortable with pharmacist activities closely linked to drug products than responsibilities associated with monitoring and optimization of patient outcomes. Medication education (96.6%) and drug knowledge (90%) were practically unanimously recognized as abilities expected of pharmacists, but consultative roles, such as assisting in drug regimen design were less acknowledged. They proposed pharmacist spend more time with physicians attending joint meetings or education events to help advance acceptance of pharmacists in patient-centered care at this site.

**Conclusions:**

Physicians had low comfort and expectations of patient-oriented pharmacist roles but were not threatened to learn more about these capabilities or explore enhanced collaboration in patient care.

## Background

Qatar is a gas- and oil-rich Arab emirate occupying a small peninsula in the Persian Gulf with a predominantly expatriate population. Qatar boasts an emerging economy and is recently devoting significant resources to develop its health care infrastructure [1,2]. Like other high-income countries, Qatar courts professionals from predominantly lower income countries to meet the demands of health service delivery [3-5]. Until only recently, there has been no local training of health professionals (the first domestically trained medical students (n = 15) graduated in 2008) and so the medical group is a heterogeneous one with physicians having studied and worked in a myriad of other settings abroad. Just as physician group multeity has been identified, backgrounds of pharmacists are similarly diverse. The first domestically trained pharmacists graduated in 2011, and like medicine, classes are small (n < 25); all pharmacists currently practicing in Qatar trained abroad [6,7].

Qatar Petroleum (QP) is the nation’s largest private employer and provides comprehensive medical care at primary care clinics throughout the country. All medications dispensed at QP pharmacies must be ordered by a physician; however, a collaborative drug therapy management model has been developed whereby pharmacists are pre-authorized to provide certain medications directly to patients. These medications are considered “over-the-counter” (OTC) in many countries based on a relative wide margin of safety and consumer-directed labeling. Direct consultation to pharmacists for OTC medication is anticipated to increase health service efficiency at QP. In this context, pharmacists are uniquely trained to provide professional guidance in selection of appropriate drug therapy and identify circumstances under which a physician should be consulted before patients embark upon independent self-care [8,9].

Potential barriers must be considered before this patient service can be successfully implemented. Physicians in Qatar may not always recognize how pharmacists function or understand how they assure safe medication use. Contemporary pharmacy practice is incompatible with a strict historic model of physicians diagnosing and prescribing while pharmacists compound and dispense [10]. Prior research in this care setting to determine acceptance of pharmacist-guided OTC self-care demonstrated patients were familiar with pharmacist roles in this regard and described a desire to use such a service [11]. Still, practical operation hinges on physician understanding of pharmacists’ skills and knowledge and willingness to expand shared patient care responsibilities. The objective of this study is to characterize physician perceptions of pharmacists and their roles in this primary care setting in Qatar.

## Methods

This cross-sectional observational study involved the survey of all physicians employed with QP (n = 80). A questionnaire was developed following comprehensive review of English language literature in pertinent electronic databases using combinations of predetermined key words. Retrieved reports were evaluated and adapted and a draft questionnaire was reviewed for face and content validity and piloted by 2 pharmacists and 2 physicians. The final survey was ultimately comprised of 19 questions encompassing physician demographics and broad domains assessing experiences and expectations with pharmacist services, comfort with pharmacist patient care roles and questions specific to pharmacist-guided patient OTC self-medication (Additional file 1: Appendix). The questionnaire was translated into Arabic according to a standardized process [12]. It was formatted and administered electronically to the physicians who would require approximately 20 min to complete and submit anonymous responses. Ethics approval was obtained from the Qatar University Institutional Review Board.

Descriptive and inferential statistics were analyzed using SPSS for Windows standard version release 17.0 (SPSS Inc., Chicago, Illinois).

## Results

Sixty-two physicians (77.5%) responded to the survey. The median age was between 40 and 50 years and almost two-third were men (64.5%). Fourteen different nationalities were represented with over half from South East Asia (India, 46.8% and Pakistan, 16.1%.), followed by the Middle East (17.7%), Europe (9.7%), North America (6.5%) and other countries (3.2%). Regions where physicians obtained their highest medical qualification were South East Asia and Europe (each 33.9%), followed by the Middle East (24.2%), North America (1.6%) and other individual countries (6.4%). Almost half (46.8%) of physicians had obtained their highest medical qualification within the past decade.

When asked to assess their comfort with specific pharmacist roles in patient care, physicians rated more highly activities closely linked to drug products (e.g. 67.8% and 75.5% were completely comfortable with pharmacists providing patients medication-related education or detecting and preventing prescription errors, respectively) than responsibilities associated with therapy optimization and monitoring of patient outcomes (e.g. 29% were uncomfortable with pharmacists recommending changes in therapy to them when patient outcomes were not achieved).

Physician expectations of pharmacist responsibilities were also assessed. Patient medication education and drug knowledge were practically unanimously recognized by physicians (96.6% and 90%, respectively) as abilities expected of pharmacists, but consultative roles, such as assisting in drug regimen design or change according to patient response were less acknowledged.

Physicians described pharmacists to be reliable sources of drug product information (75.3%), experienced pharmacists counseling patients on safe and effective medication use (70.4%), but were less familiar with pharmacists inquiry into expectations or desired drug therapy outcomes for patients (38.8%). Over half (55.6%) reported having a relationship with at least one pharmacist with whom to consult on prescribing matters. In fact, collaborative attitudes towards interprofessional care were high. Physicians were not threatened to learn more about pharmacist capabilities and explore enhanced participation in patient care. However, cultural issues related to professional boundaries emerged when respondents offered perceived obstacles to working with pharmacists. They proposed that spending more time with physicians attending joint meetings or education events could help advance pharmacist acceptance in patient-centered care at this site.

When results were stratified, no differences were found according to nationality, gender, or years in practice.

## Discussion

Physicians at this private care setting in Qatar were most comfortable with product-related roles of pharmacists when compared to patient-oriented activities. Other investigators have found level of physician acceptance with specific clinical pharmacy services (e.g. advising physicians on medication selection and monitoring drug regimens) was related to the exposure they had to these contemporary pharmacy-services [13]. Clinical pharmacy programs are maturing with small numbers of pharmacists forming part of asthma and diabetes management programs at a few QP medical sites, therefore, interprofessional interactions are shifting from predominantly reactive ones, as in prescription order clarification [14]. Exposure to pharmacists in decentralized roles, as in these chronic disease programs, could augment appreciation of pharmacist broader skills and contribution to care. Indeed, a survey of physicians at the major tertiary care center in Qatar, exposed to pharmacists at the patient point-of-care, were more accustomed with receiving pharmacist input in the design and monitoring of drug therapy regimens than physicians in our study [15].

Younger physicians from North American backgrounds have expressed higher expectations of pharmacy practice and collaboration when compared to senior colleagues, but we were unable to detect this in our report [16,17]. Instead, we observed a non-significant trend of longer work experience at QP with higher physician expectations of pharmacist roles, which may be due to familiarity with local capabilities. The value physicians place on specific aspects of pharmacist activity and perception of pharmacist competence in carrying out these functions are also reported to influence physician views [16,18]. In one recent study, general practitioners level of support of pharmacists’ roles in medication management was high for technical and safety functions (e.g. checking prescriptions) and progressively diminished for more “clinical” services (advising patients on medicine’s potential benefit, monitoring effectiveness) [19].

While most published reports of physician attitudes come from countries where forward-moving pharmacy practice roles in direct patient care activities have been advancing for decades, a few surveys have been reported in the region. Hospital physicians in Kuwait, Jordan, and Sudan were comfortable with pharmacists detecting and preventing prescription errors and providing patient education, but were uncomfortable with pharmacists recommending drug therapy to patients, even for minor ailments [20-22]. Medical authority is conceived differently in this region such that Arab patients tend to automatically defer to the doctor’s authority, challenging the prevailing patient-centered health models promoted in Western societies. Indeed, information tends to flow from the “top down” and a high degree of deference is afforded physicians within Arab contexts, not only by patients, but other health care providers [23]. Such attitudes, beliefs and cultural customs may serve as initial barriers to pharmacists’ ability and motivation to assume partnership in medication-related decision making. Multinational health providers must capitalize on their diverse knowledge and skills, but also embrace contemporary collaborative models of care in order to achieve national health care reforms in this country [24].

Our study has several limitations. While the response rate was high, the sample was small therefore restricting generalizability of our findings. It is unclear what specific exposure to interdisciplinary care these physicians had during medical training or prior employment experiences and so their views may be different from practitioners working in closer proximity with pharmacists and multidisciplinary teams in a tertiary care hospital, for example. We did not conduct a parallel inventory of pharmacist training and it is possible that skills and confidence encompass a broad spectrum within this practice site and account somewhat for reduced experience and expectations of physicians for certain patient-centered roles.

## Conclusion

Physicians had low comfort and expectations of patient-oriented pharmacist roles but were not threatened to learn more about these capabilities or explore enhanced collaboration in patient care.

## Competing interests

The authors report no perceived or actual conflict of interests.

## Authors’ contributions

KW devised the project concept. All authors conducted the literature review. AB and AH developed and translated the questionnaire. AB and AH administered the questionnaire. All authors synthesized and analyized the data. KW drafted the manuscript. All authors read and approved the final manuscript.

## Funding

This work has been presented at the Dubai International Pharmaceuticals and Technology Exhibition and Conference (DUPHAT) in March 2011.

## Authors’ contributions

KW devised the project concept. All authors conducted the literature review. AB and AH developed and translated the questionnaire. AB and AH administered the questionnaire. All authors synthesized and analyized the data. KW drafted the manuscript. All authors read and approved the final manuscript.

## Supplementary Material

Additional file 1 Appendix. Click here for file
