# Supplementary material for: Physician perceptions of pharmacist roles in a primary care setting in Qatar
Source: Global Health. 2012 May 31;8:12. doi: 10.1186/1744-8603-8-12 (PMC3475112; doi:10.1186/1744-8603-8-12)
Supplement: Additional file 1 — Appendix. [file 1744-8603-8-12-S1.pdf]

جامعة قطر
QATAR UNIVERSITY

APPENDIX – PHYSICIAN QUESTIONNAIRE

1. **Please indicate your gender**: ☐ Male ☐ Female

2. **Please select the age group to which you belong**: ☐ < 30 ☐ 30-40 ☐ 40-50 ☐ 50-60 ☐ >60

3. **Please indicate your nationality**: ___________________________

4. **Please select the Highest Family Medicine Qualification obtained:** ☐ Board certification ☐ Masters ☐ Postgraduate diploma

5. **Please indicate the country where highest medical qualification obtained:** ___________________________

6. **Please select the year group in which you obtained your highest medical qualification:**

   Year of ☐ 2000-2009 ☐ 1990-1999 ☐ 1980-1989 ☐ 1970-1979 ☐ <1970

7. **Please indicate the number of years you have been working in primary health care:** ☐ < 5 ☐ 5-10 ☐ 11-15 ☐ > 15

8. **Please indicate the number of years you have been working at QP Medical Services:** ☐ < 5 ☐ 5-10 ☐ 11-15 ☐ > 15

9. **Please estimate the <u>annual average</u> of approved CPD / CME hours you have participated in over the past 5 years:** _____________

10. **In an average month, please estimate the frequency of your interactions with a pharmacist:**
    ☐ EVERY DAY ☐ MORE THAN ONCE/WEEK ☐ ONCE A WEEK
    ☐ MORE THAN ONCE/MONTH ☐ ONCE A MONTH ☐ LESS THAN ONCE/MONTH

11. **Please rank the following according to the most frequent type of interaction <u>you</u> initiate with a pharmacist:**

(Start at **1** for **most frequent** and finish with 5 or 6 for least frequent)

__ Questions about drug availability at QP
__ Questions about drug alternatives available at QP
__ Questions about drug dosages
__Questions about medication side effects
__Questions about drug interactions
__Other: ____________________________

12. **Please rank the following according to the most frequent type of interaction <u>a pharmacist</u> initiates with you:**

(Start at **1** for **most frequent** and finish with 5 or 6 for least frequent)

__ Clarifying a prescription order (drug dosing or drug choice)
__ Requesting a prescription change due to patient medication allergy
__ Requesting a prescription change due to unavailability of medication at QP
__Discussions about patient non-adherence to drug therapy
__Requests for more patient history
__Other: ____________________________

جامعة قطر
QATAR UNIVERSITY

13. Please rate your **<u>comfort</u> with pharmacist roles** described below.
Select the number which best applies to your level of comfort.

**PHARMACIST ROLES**	**COMPLETELY COMFORTABLE**	**SOMEWHAT COMFORTABLE**	**UNCOMFORTABLE**
Providing medication-related patient education	1	2	3
Providing disease-related patient education	1	2	3
Suggesting use of non-prescription medication(“over-the counter” (OTC)) to patients *(e.g. paracetamol, antacids)*	1	2	3
Suggesting use of prescription medications to patients (*e.g. antibiotics, cardiovascular therapy)*	1	2	3
Suggesting patient use of prescription medication to the physician	1	2	3
Treating minor illnesses (*e.g. headache, dyspepsia)*	1	2	3
Detecting and preventing prescription errors	1	2	3
Designing drug therapy regimens (drug selection, dose, interval, length of therapy)	1	2	3
Monitoring safety of drug therapy regimens (i.e. side effect assessment)	1	2	3
Monitoring outcomes of drug therapy regimens and plans (i.e. has patient achieved desired blood pressure)	1	2	3
Recommending changes to drug therapy regimens to physicians when outcomes are not achieved	1	2	3

14. The following is a list of statements pertaining to your **<u>expectations</u> of pharmacist roles**. Please select your level of agreement with these statements.

**PHARMACIST ROLES**	**STRONGLY AGREE**	**AGREE**	**NEITHER AGREE OR DISAGREE**	**DISAGREE**	**STRONGLY DISAGREE**
I expect pharmacists to take personal responsibility for resolving any drug-related problems they discover involving my patients	1	2	3	4	5
I expect pharmacists to be knowledgeable drug therapy experts	1	2	3	4	5
I expect pharmacists to assist me in designing drug therapy treatment plans for my patients	1	2	3	4	5
I expect pharmacists to educate my patient about the safe and appropriate use of their medication	1	2	3	4	5
I expect pharmacists to monitor my patients' response to drug therapy and let me know if a patient encounters any drug-related problems	1	2	3	4	5
I expect pharmacists to assist my patients in selecting the appropriate non-prescription ("OTC") medications	1	2	3	4	5
I expect pharmacists to know the specific indication for each drug I prescribe	1	2	3	4	5
I expect pharmacists to be available to me for consultation when I see patients *(e.g. during a clinic visit or appointment)*	1	2	3	4	5
I expect the pharmacists to take reasonable steps to ensure that my patients have their medications refilled on time	1	2	3	4	5
I expect the pharmacist to recommend changes to drug therapy regimens to the physician when outcomes are not achieved for patients	1	2	3	4	5

15. The following is a list of statements pertaining to your **experience with pharmacists**.
Please select your level of agreement with these statements.

	STRONGLY AGREE	AGREE	NEITHER AGREE OR DISAGREE	DISAGREE	STRONGLY DISAGREE
In my experience, pharmacists are reliable source of general drug information (i.e. facts about drug which can be found in standard references)	1	2	3	4	5
In my experience, pharmacists are reliable source of clinical drug information (i.e. information regarding the clinical use of drug in specific patient situations)	1	2	3	4	5
In my experience, pharmacists routinely counsel my patients regarding the safe and appropriate use of their medications	1	2	3	4	5
In my experience, when pharmacists discover problems with my prescription, they privately inform me	1	2	3	4	5
In my experience, pharmacists routinely inform me about more cost-effective alternatives to the drugs I prescribe	1	2	3	4	5
In my experience, pharmacists frequently ask me to clarify for them the drug therapy objectives or desired outcomes I have in mind for my patients	1	2	3	4	5
In my experience, pharmacists frequently let me know that my patients have experienced some problems with their medication	1	2	3	4	5
In my experience, pharmacists appear willing to take responsibility for finding solutions to any drug-related problems they discover	1	2	3	4	5
I have a relationship with one or more pharmacists wherein I collaborate with them to determine and adjust my patient's drug therapy regimen	1	2	3	4	5

16. The following is a list of statements describing some negative experiences with pharmacists. Please describe whether you have ever encountered such a behaviour.

**Experience**	**Yes**	**No**
The pharmacist reportedly tried to be helpful by educating the patient, but actually scared patient and the patient no longer wished to take the drug		
The pharmacist gave the patient the wrong drug or wrong dosage		
The pharmacist reportedly made inappropriate comments about a prescribed medication in the presence of a patient		
The pharmacist reportedly made inappropriate comments about a prescriber in presence of a patient		
The pharmacist refilled prescription not indicated for refill		
The pharmacist refused to order a medication not in the formulary		
The pharmacist changed the original directions on a prescription refill (prescriber’s order) inappropriately		

17. Please describe the most common barrier(s) or obstacle(s) for multidiscipline (interprofessional) collaboration among pharmacists and physicians:

QATAR UNIVERSITY

18. The following is a list of statements pertaining to your **collaboration with pharmacist.** Please select your level of agreement with these statements.

	STRONGLY AGREE	AGREE	NEITHER AGREE OR DISAGREE	DISAGREE	STRONGLY DISAGREE
I believe interprofessional collaboration may involve a risk to my profession and image as a physician	1	2	3	4	5
I am willing to accept the changes and perceived risks involved in developing collaborations with pharmacists	1	2	3	4	5
I think expanding pharmacist roles in patient care will prevent me from providing the best care to my patients as the physician	1	2	3	4	5
Physician familiarity with pharmacist abilities could lend support to a physician’s willingness to accept a pharmacist’s input.	1	2	3	4	5
Physician respect for pharmacist abilities could lend support to a physician’s willingness to accept a pharmacist’s input.	1	2	3	4	5

19. Please indicate any comments/ideas/improvements needed for pharmacist roles in patient-centered care:

20. You have reached the end of the survey. Thank you for your time.
